# Exploration of patient plasma exosomes as biomarkers for predicting lung cancer brain metastasis

**DOI:** 10.3389/fmed.2025.1687866

**Published:** 2025-12-03

**Authors:** Lingyun Ye, Xinnan Hu, Shen Chen, Hai Wang, Juanjuan Li, Lei Wang, Guanghui Gao, Xiaoxia Chen, Xiao Song

**Affiliations:** 1 Department of Oncology, Shanghai Pulmonary Hospital & Thoracic Cancer Institute, Tongji University School of Medicine, Shanghai, China; 2 Department of Endoscopy Center, Shanghai Pulmonary Hospital, Tongji University School of Medicine, Shanghai, China; 3 Department of Thoracic Surgery, Shanghai Pulmonary Hospital, Tongji University School of Medicine, Shanghai, China

**Keywords:** exosome, miRNA, biomarker, lung cancer brain metastasis, lung cancer

## Abstract

**Background:**

Many studies have found that exosomes have numerous advantages in the early diagnosis of tumors. We detected and analyzed plasma exosomes from lung cancer patients to identify potential biomarkers that could predict brain metastasis.

**Method:**

The total RNA of plasma exosomes of advanced lung cancer patients was extracted and sequenced. The BLAST software was used to align the predicted target gene sequence against the GO and KEGG databases, thereby acquiring annotation details for the target genes. The selected exosomal miRNAs and short-chain fatty acids were subjected to diagnostic performance validation analysis.

**Results:**

Exosomal miR-223-3p, miR-27a-3p, and miR-27b-3p were significantly increased in the plasma exosomes of lung cancer patients with brain metastasis. The concentrations of isobutyric acid (IBA), valeric acid (VA), isovaleric acid (IVA), and acetic acid (AA) were markedly elevated in the plasma exosomes of lung cancer patients with brain metastasis. Spearman correlation analysis revealed that both miR-27a-3p and miR-27b-3p had significant associations with IVA and VA. A multi-biomarker model combining the selected exosomal miRNAs with metabolic molecules could improve the diagnostic performance with an AUC of 0.927.

**Conclusion:**

Plasma exosomal miR-223-3p, miR-27a-3p, miR-27b-3p, and IBA, IVA, VA, and AA in advanced patients are closely associated with brain metastasis and have the potential to act as biomarkers for predicting brain metastasis in lung cancer patients.

## Introduction

Nowadays, with advancements in diagnostic and treatment technologies and early detection of asymptomatic lesions, the survival duration of lung cancer patients has notably increased (1). However, concurrently, an increasing number of patients succumb to tumor metastasis, especially brain metastasis from lung cancer.

Brain metastasis is notably prevalent among lung cancer patients, representing at least half of all brain metastases (2, 3). The onset of brain metastasis in lung cancer signals advanced tumor progression, carrying a grim prognosis. Non-small cell lung cancer (NSCLC) stands as the predominant histological subtype of lung cancer, encompassing 80–85% of all lung cancers. At the point of initial diagnosis, 20% of NSCLC patients already exhibit brain metastasis, with 40–50% progressing to brain metastasis as the disease unfolds (4–6). Lung cancer’s brain metastasis can be categorized into leptomeningeal metastasis (LM) and brain parenchymal metastasis (BM), with the latter being more frequent. Certain studies suggest that the site of brain metastasis in lung cancer correlates with blood flow and tissue distribution: 80% is localized within the cerebral hemisphere, 15% in the cerebellum, and 5% in the brainstem (1). Brain metastasis from lung cancer can manifest various neurologically related symptoms, such as headaches, seizures, cognitive deficits, behavioral alterations, and gait disturbances, all of which significantly degrade patient quality of life.

Presently, predictions concerning the likelihood of brain metastases in lung cancer patients rely solely on clinical and pathological variables. In an examination of 182 lung adenocarcinomas with remote metastases, Hung et al. (7) discerned a significant association between micropapillary histological subtypes and brain metastases (*p* = 0.01). However, the heterogeneity of data on predictive parameters diminishes their clinical utility. Using standard clinical and pathological factors (e.g., primary tumor status, tumor histology, lymph node status, and patient age) to identify patients with the highest propensity for brain metastases might be unreliable, owing to their minimal hazard ratios and unspecified prognostic elements (8). Hence, there is an immediate need for a more dependable methodology to discern patients at risk of developing brain metastases.

In recent years, exosomes derived from tumor cells have attracted substantial interest, functioning as pivotal agents of intercellular communication, significantly influencing brain metastasis formation. Exosomes are extracellular vesicles with typical diameters ranging from 30 to 150 nm (9–11) and can be detected in diverse bodily fluids, including blood, urine, and saliva (12). Prior research indicates that tumor-derived exosomes often transport crucial signaling molecules, such as miRNAs, which are pivotal in tumor signal transduction of the microenvironment. They influence the genesis and progression of brain metastases and have potential as biomarkers for early diagnosis and prognostic evaluation of lung cancer.

## Materials and methods

### Patients

This study encompassed advanced lung cancer patients admitted to Shanghai Pulmonary Hospital from January 2019 to December 2022. All patients were pathologically confirmed as NSCLC and were categorized as stage IV patients according to the eighth edition of the lung cancer TNM staging. Brain metastasis evaluations were conducted using enhanced MRI. Patients with multiple active primary malignant tumors were omitted. The research was approved by the Ethics Committee of Tongji University Shanghai Pulmonary Hospital. All participants provided informed consent for peripheral blood collection for further exosome analysis.

### Extraction and identification of the patient’s plasma exosomes

Plasma exosome was purified using Exosupur^®^ columns (Echobiotech, China). The plasma underwent filtration through a 0.8 μm filter. The filter was then rinsed with 250 μL of PBS using a syringe, adjusting the filtrate volume to approximately 1 mL, and the filtrate was maintained at room temperature. The exclusion column was positioned on its designated rack and pre-rinsed with 0.1 M PBS. Once the column achieved equilibrium and the fluid on its upper sieve plate drained, the pretreated plasma was introduced. A collection tube was placed beneath the exclusion column, PBS was added for elution, and the exosomes were subsequently gathered. All retrieved exosomes were transferred to a 100 kDa ultrafiltration tube and centrifuged at 4,000 g for 2 min, reducing the residual liquid to roughly 200 μL. Post exosome collection, the exclusion column was flushed with 0.1 M PBS, followed by a rinse with 5 mL of 20% ethanol. To seal the column’s upper and lower outlets, 1 mL of 20% ethanol was introduced.

### Tracking analysis of exosomes

The dimensions and particle concentration of the exosomes were ascertained using ZetaView PMX 110 (Particle Metrix, Meerbusch, Germany, Beijing Echobiotech Co., Ltd.). The NTA software (ZetaView 8.02.28) facilitated particle trajectory analyses, yielding detailed size and concentration of the exosomes.

### Detection of exosomes by transmission electron microscope

The exosome suspension was incubated with the copper mesh for 2 min at room temperature, followed by a sterile distilled water rinse. Negative staining was performed with a uranyl acetate solution for 1 min. The sample was dried beneath an incandescent lamp for 2 min. Transmission electron microscopy facilitated the observation and imaging of the exosomes.

### RNA extraction of exosomes from the plasma of patients

In accordance with the kit instructions, total RNA was isolated and purified from plasma exosomes employing the miRNeasy Plasma/Plasma Advanced Kit (Qiagen, catalogue number 217204). To a 200 μL plasma sample, 700 μL of QIAzol lysate solution was added, followed by incubation for 5 min at ambient temperature. Following this, 140 μL of chloroform (trichloromethane) was introduced to each sample tube. The tubes were then incubated at room temperature for 2–3 min and subsequently centrifuged at 12,000 g for 15 min at 4 °C. The supernatant was decanted into a new EP tube, mixed with 1.5 times its volume of absolute ethanol, transferred to an RNA adsorption column in the collection tube, and centrifuged at 8,000 g for 15 s at ambient temperature. The wash solution was added to the centrifuge column, which was then centrifuged, and the filtrate discarded. The RNA adsorption column was relocated to a new 2 mL collection tube and spun at 12,000 g for 2 min. Subsequently, the column was moved to a fresh 1.5 mL EP tube, where 50 μL of RNase-free ddH_2_O was added before centrifugation at 12,000 g at room temperature. The centrifugation column was finally discarded, yielding exosomal RNA.

### miRNA library building and sequencing analysis

For constructing small RNA libraries, an input quantity of 3 ng of RNA was obtained from each sample and used for RNA sample preparation. The sequencing libraries were subsequently generated using the QIAseq miRNA Library Kit (manufactured by Qiagen, located in Frederick, MD), adhering to the instructions provided by the manufacturer. The 3′ ligation reaction solution and the 5′ ligation reaction solution were prepared as instructed, and reverse transcription was conducted post-incubation. A volume of 2 μL of QIAseq miRNA NGS Reverse Transcription Initiator was introduced into the reaction tube. The reverse transcription reaction mixture was assembled on ice. After combining QIAseq Beads with QIAseq miRNA NGS Bead Binding Buffer, 400 μL of QIAseq Beads was dispensed into the microcentrifuge tube and isolated using a magnetic stand. The beads underwent successive treatments with QIAseq miRNA NGS Bead Binding Buffer. 143 μL of QMN beads was added to each cDNA reaction tube, incubated at room temperature for 5 min, followed by magnetic separation. The DNA was eluted in sterile water and transferred to a fresh tube. The library amplification reaction was set up on ice, and PCR was performed as instructed. 1 μL of the miRNA sequencing library was evaluated on an Agilent Bioanalyzer system utilizing the High Sensitivity DNA Analysis Kit (or chip). Qualified libraries underwent sequencing on the Illumina NovaSeq 6000 platform, and the resulting data were subsequently analyzed.

### miRNA analysis

The expression matrix, which documented the quantified unique molecular identifier (UMI) counts for miRNAs, was normalized to transcripts per million (TPM) values. Relative log expression values were derived from the normalized data using the EdgeR computational package. Differential expression analysis between the two groups was conducted using the Mann–Whitney *U* test, with a significance threshold of *p* < 0.05 and an absolute log₂-transformed fold-change (|log₂FC|) cutoff of ≥0.58.

Potential miRNA target genes included the miRNAs identified using miRanda and RNAhybrid. Functional enrichment analysis using Gene Ontology (GO) terms was performed on the target genes of differentially expressed miRNAs using the topGO R packages. KEGG pathway enrichment was analyzed using a Python program, KOBAS (43). Automated genome annotation and pathway identification were carried out using KEGG Orthology as a controlled vocabulary.

### Reverse transcription and qPCR quantitative analysis

Total RNA was subsequently reverse transcribed into complementary DNA (cDNA) using the PrimeScript^™^ RT Reagent Kit (Perfect Real Time) (TAKARA, Cat. No. RR037A). Target gene expression levels were quantified via TaqMan^®^ probe in real-time qPCR. Each PCR reaction utilized 2 μL of cDNA as the template. Primer and probe sequences are detailed in the accompanying table.

Exosomal RNA from patient plasma was thawed, combined, centrifuged, and set on ice. After amalgamating and spinning the prepared RT-preMix, reverse transcription was performed on a PCR machine (37 °C for 60 min, 85 °C for 5 s, and then maintained at 4 °C). The resultant cDNA was promptly employed for qPCR quantitative analysis. Samples were gently mixed, briefly centrifuged, and held on ice. The formulated qPCR PreMix was apportioned into a 96-well plate for the qPCR reaction, sealed with film, spun in a centrifuge, and the qPCR program was initialized. For result analysis, the relative quantity approach was used based on the Ct value, using the formula: 2^−ΔΔCT^. A *t*-test was conducted to compare the miRNA expression levels in the two groups.

### Quantification of short-chain fatty acids

Fecal metabolite quantification was performed by EchoBiotech Co., Ltd., Beijing, P. R. China. Exosome samples were retrieved from the −80 °C freezer and thawed on ice. A volume of 100 μL of a 0.5% v/v phosphoric acid solution was introduced into the sample tube. The resulting mixture was vortexed for 3 min. A volume of 150 μL of an MTBE solution (which included an internal standard) was introduced into the tube. The mixture was then vortexed for 3 min and subsequently ultrasonicated for 5 min. The mixture was then centrifuged at 12,000 r/min for 10 min at 4 °C. The supernatant was retrieved and then analyzed by GC-MS/MS. An Agilent 7890B gas chromatograph, which was connected to a 7000D mass spectrometer and equipped with a DB-FFAP column (30 m in length, 0.25 mm internal diameter, and a 0.25 μm film thickness, manufactured by J&W Scientific in the United States), was employed for GC-MS/MS analysis of SCFAs. Helium was used as a carrier gas at a flow rate of 1.2 mL/min. Injection was performed in split mode with a 2 μL injection volume. The oven temperature was held at 90 °C for 1 min, raised to 100 °C at a rate of 25 °C/min, then raised to 150 °C at a rate of 20 °C/min, held for 0.6 min, raised to 200 °C at a rate of 25 °C/min, and held for 0.5 min after running for 3 min. Each sample was analyzed in the multiple reaction monitoring operational mode. We configured the injector inlet temperature to 200 °C and the transfer line temperature to 230 °C.

### Statistical analysis

All statistical tests were conducted utilizing SPSS 22.0. The nonparametric test was used to contrast age differences across the groups, and the chi-square test was used to analyze the differences in other clinical features. Define *p* < 0.05 as statistically significant.

A multifactor logistic regression analysis was performed to examine two groups that showed statistical significance, and a predictive model was subsequently constructed. The receiver operating characteristic (ROC) curve was used to determine the predictive worth of the regression model.

An analysis of ROC curves was performed, and the AUC (area under the ROC curve) was determined using the pROCR package.

## Results

### Identification of exosomes in the plasma of lung cancer patients

**Figure 1:**
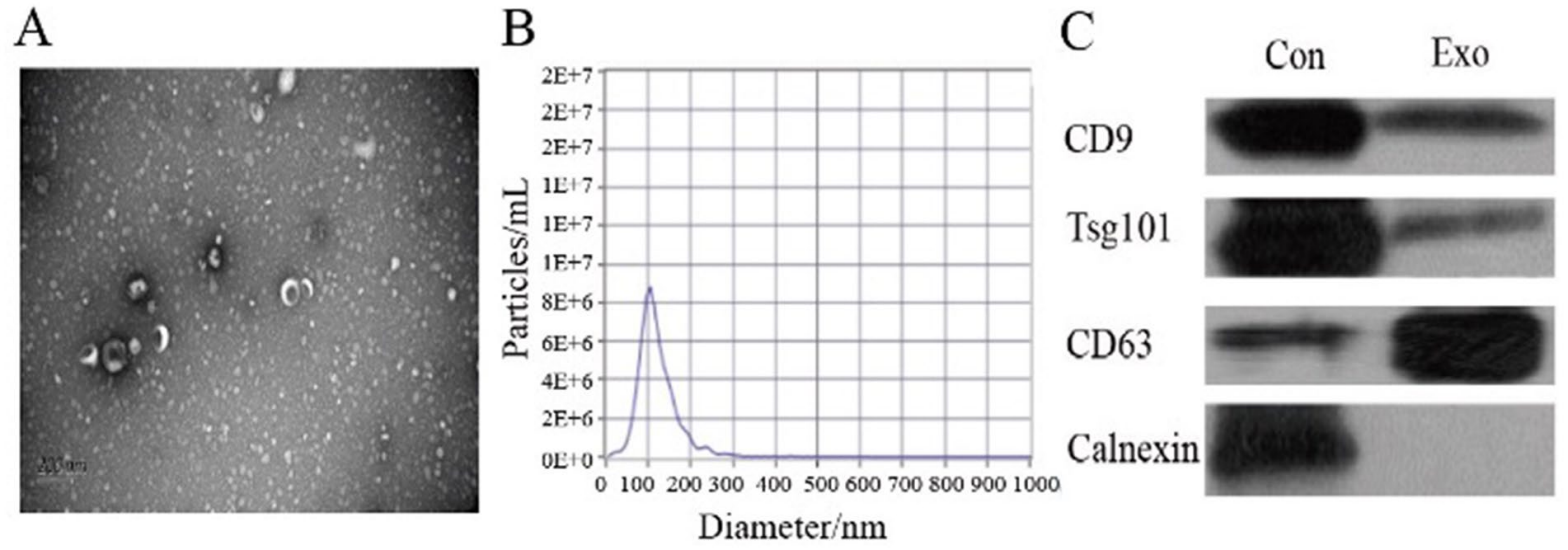
Identification of plasma exosomes in advanced lung cancer patients. **(A)** Transmission electron microscopy revealing the structure of the plasma exosomes isolated from patients. **(B)** Particle size measurement of the identified plasma exosomes. **(C)** Western blot analysis illustrating the detection results of plasma exosome components. Con: positive control; Exo: the patient’s plasma exosomes.

A total of 26 patients with advanced lung cancer were enrolled, which included 14 patients with brain metastasis and 12 patients without brain metastasis. Plasma from each patient was collected and used for exosome extraction. Table 1 depicts the clinical data of the patients. Plasma exosomes from lung cancer patients were identified using transmission electron microscopy, particle size measurement, and Western blot. Transmission electron microscopy results revealed that the patients’ plasma exosomes showed a typical tea tray-like appearance with a visible membrane structure (Figure 1A). Particle size measurements showed that these exosomes were approximately 100 nm in size (Figure 1B). Western blot experiments confirmed the expression of positive protein markers CD9, Tsg101, and CD63, while the negative protein marker calnexin was not detected (Figure 1C).

**Table 1:** Clinical information for patients with advanced lung cancer enrolled in the exosomes identification.

		Brain metastasis		
Characteristic	Overall	No	Yes	*p*
	26	14	12	
Age [median (IQR)]	62 [54, 67]	61 [54, 68]	62 [57, 67]	0.9589
Gender (%)				
Female	3 (11.54)	2 (14.29)	1 (8.33)	1.000
Male	23 (88.46)	12 (85.71)	11 (91.67)	
Smoke history^a^ (%)				
Mild	2 (7.69)	2 (14.29)	0 (0.00)	0.2161
Moderate	20 (76.92)	11 (78.57)	9 (75.00)	
Severe	4 (15.38)	1 (7.14)	3 (25.00)	
Histological type (%)				
Adenocarcinoma	23 (88.46)	13 (92.86)	10 (83.33)	0.1963
Squamous	1 (3.85)	1 (7.14)	0 (0.00)	
Others	2 (7.69)	0 (0.00)	2 (16.67)	
Gene mutation (%)				
EGFR	4 (15.38)	3 (21.43)	1 (8.33)	0.4907
HER2	1 (3.85)	1 (7.14)	0 (0.00)	
KRAS	3 (11.54)	2 (14.29)	1 (8.33)	
WT	18 (69.23)	8 (57.14)	10 (83.33)	
Other metastasis (%)				
Bone metastasis	6 (23.08)	4 (28.57)	2 (16.67)	0.6343
Liver metastasis	3 (11.54)	2 (14.29)	1 (8.33)	
None	17 (65.38)	8 (57.14)	9 (75.00)	

a Smoking index (SI) = daily smoking quantity × years of smoking. SI ≤ 200: Mild smoking; 200–400: Moderate smoking; ≥ 400: Severe smoking.

### Plasma exosomal miRNA sequencing analysis of advanced lung cancer patients with or without brain metastasis

**Figure 2:**
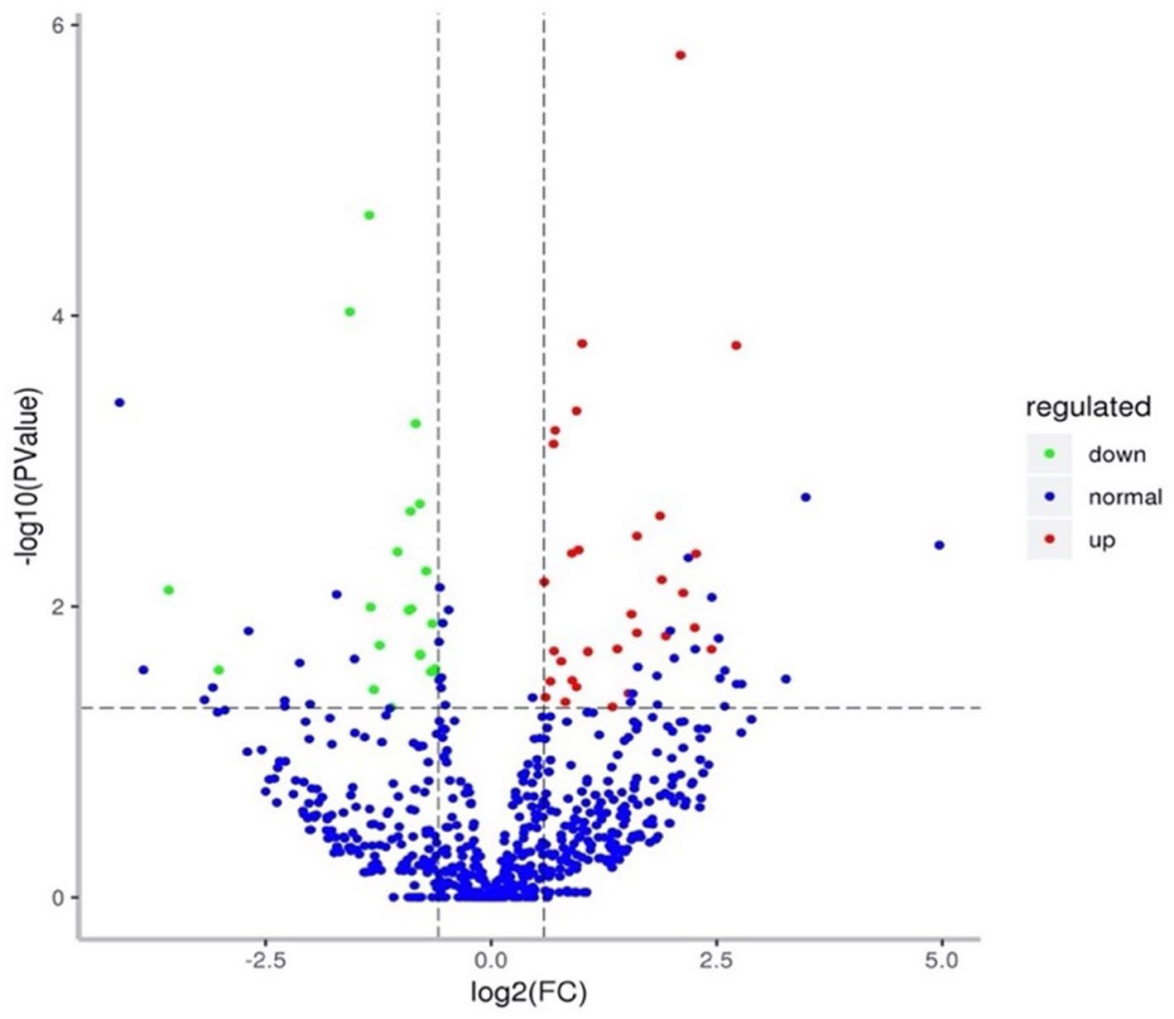
Differential expression of miRNAs in plasma exosomes of advanced lung cancer patients. Each data point denotes a specific miRNA; red points indicate upregulated miRNAs, green points indicate downregulated miRNAs, and blue points signify miRNAs with undifferentiated expression.

**Figure 3:**
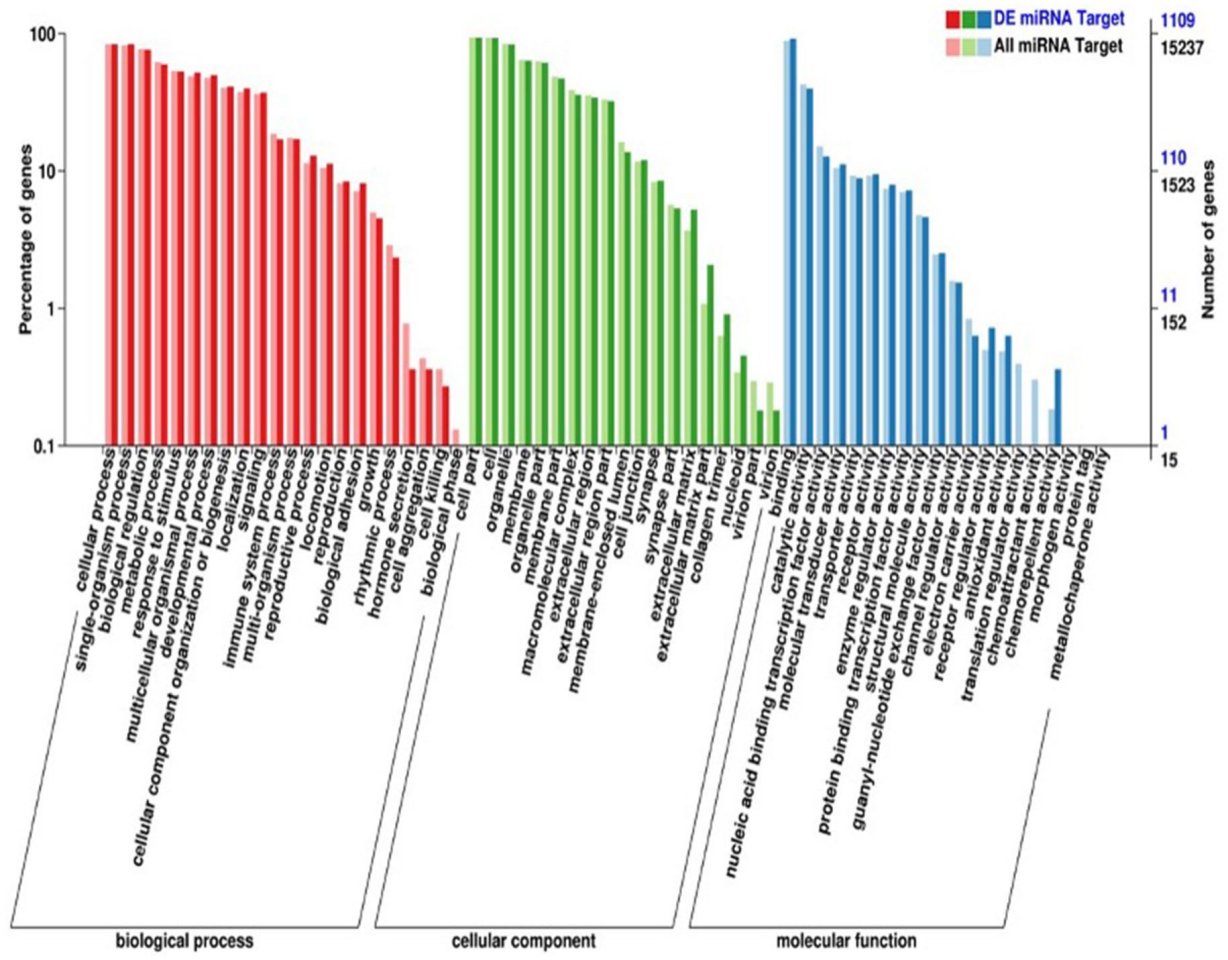
GO annotation classification of differentially expressed miRNA target genes in plasma exosomes. The horizontal axis delineates the categories listed in the GO database, whereas the vertical axis depicts the proportion and total number of genes classified in each category. The target gene functions of differentially expressed miRNAs were related to the extracellular matrix, collagen trimer, and nucleoid.

**Figure 4:**
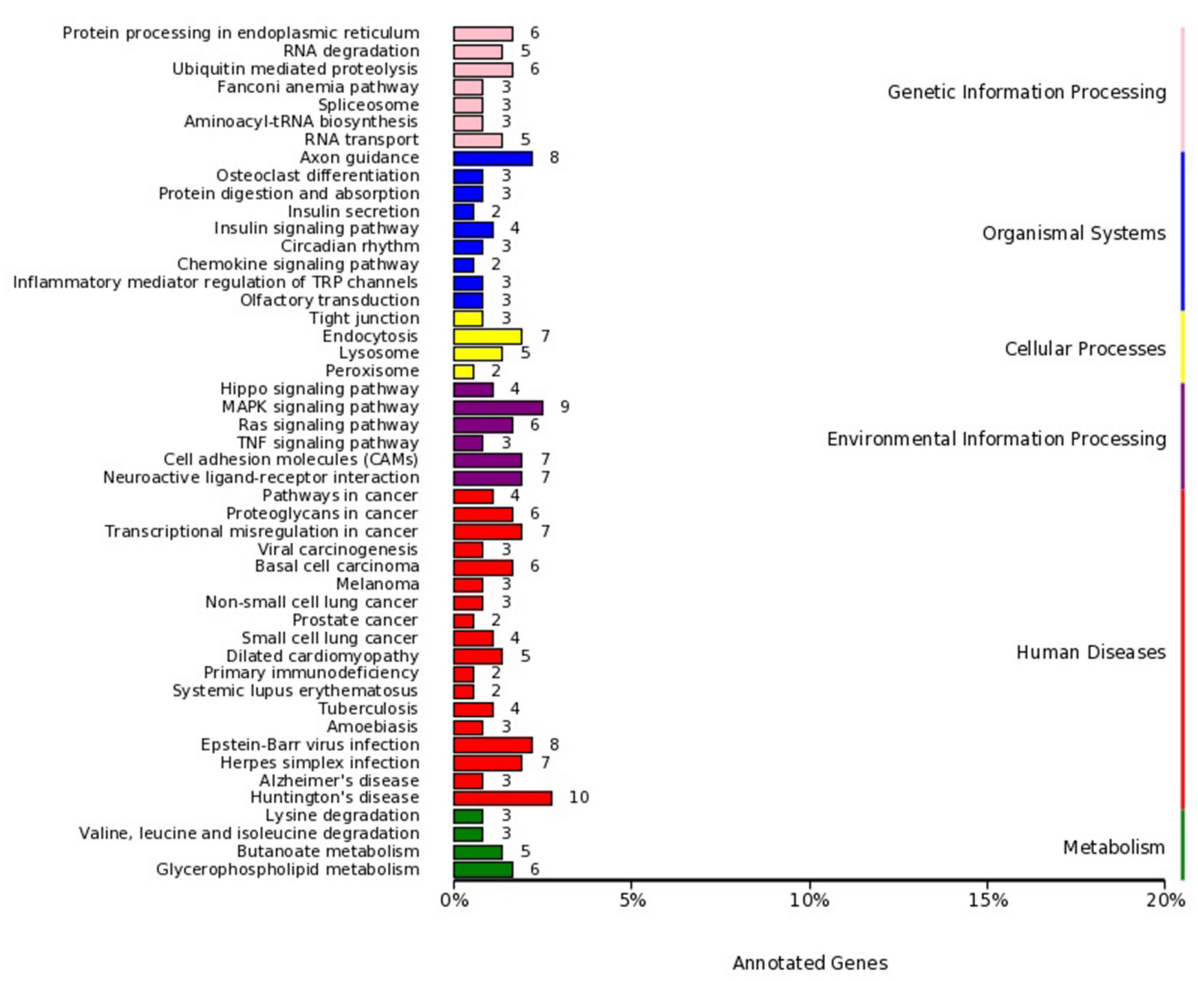
KEGG pathway classification of target genes of differentially expressed miRNAs in plasma exosomes. The vertical axis enumerates the various KEGG pathways, and the horizontal axis indicates the number and percentage of genes annotated within each respective pathway. Pink represents genetic information processing, blue represents organismal systems, yellow represents cellular processes, purple represents environmental information processing, red represents human diseases, and green represents metabolism. The target genes were linked to protein processing in the endoplasmic reticulum, ubiquitin-mediated proteolysis, RNA transport, and the regulation of cell adhesion and endocytosis.

**Figure 5:**
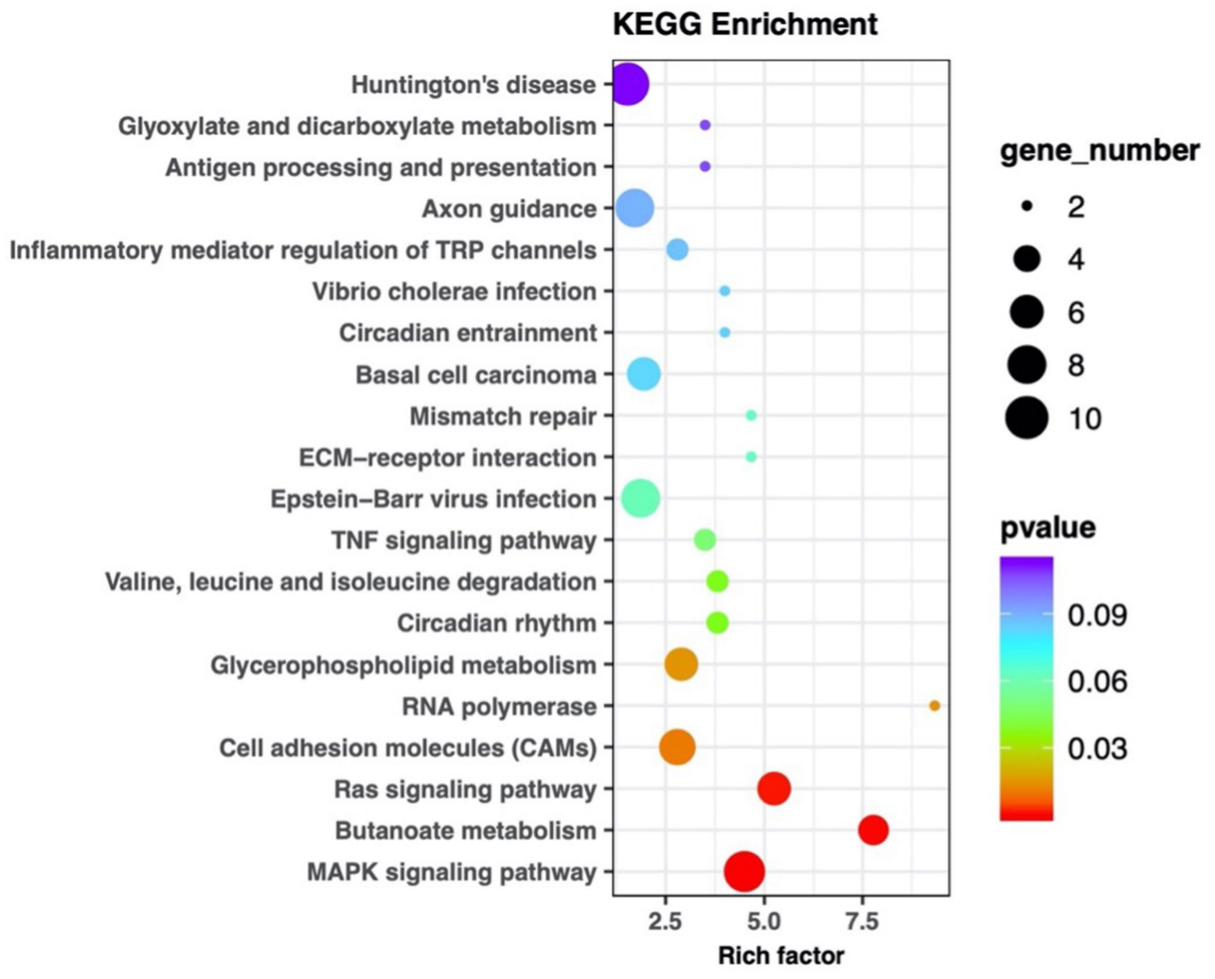
KEGG pathway enrichment scatter plot of differentially expressed miRNA target genes in plasma exosomes. Each marker within the plot represents an individual signaling pathway. The size of the dots in the graph represents the number of genes. The legend on the left specifies each pathway name, with the abscissa representing the enrichment factor. The target genes were implicated in the MAPK, Ras signaling pathways, and butanoate metabolism.

MiRNA sequencing analysis revealed a total of 892 miRNAs in the plasma exosomes of advanced lung cancer patients, regardless of the presence of brain metastasis. Of these, 873 were known, and 19 were newly predicted. Compared to late-stage lung cancer patients without brain metastasis, 31 miRNAs were significantly upregulated, and 23 were significantly downregulated in the patients with brain metastasis (Figure 2). MiRanda and RNAhybrid were used to predict the target genes of these miRNAs, and the results showed that a total of 15,615 target genes were found. BLAST software was used to match target gene sequences with the KEGG and GO databases, and the resulting annotation data of these genes were retrieved. As shown in Figure 3, the target gene functions of differentially expressed miRNAs (predicted through GO classification) were related to the extracellular matrix, collagen trimer, and nucleoid. These target genes played roles in regulating intercellular adhesion, receptor regulation, antioxidants, and other processes vital to tumor onset, growth, and metastasis. According to the KEGG classification, the target genes of the differentially expressed miRNAs within plasma exosomes derived from advanced lung cancer patients were linked to protein processing in the endoplasmic reticulum, ubiquitin-mediated proteolysis, RNA transport, and the regulation of cell adhesion and endocytosis (Figure 4). Furthermore, these target genes played a significant role in the MAPK and Ras signaling pathways, which are essential for tumor development and metastasis. These genes were also implicated in butanoate metabolism regulation, as indicated by large enrichment factors, suggesting significant differences in the butanoate metabolic pathways (Figure 5).

The vertical axis lists the various KEGG pathways, and the horizontal axis indicates the number and percentage of genes annotated within each pathway. Pink represents genetic information processing, blue represents organismal systems, yellow represents cellular processes, purple represents environmental information processing, red represents human diseases, and green represents metabolism. The target genes were linked to protein processing in the endoplasmic reticulum, ubiquitin-mediated proteolysis, RNA transport, and the regulation of cell adhesion and endocytosis.

### RT-qPCR validation of miRNAs differentially expressed in plasma exosomes of advanced lung cancer patients with or without brain metastasis

**Figure 6:**
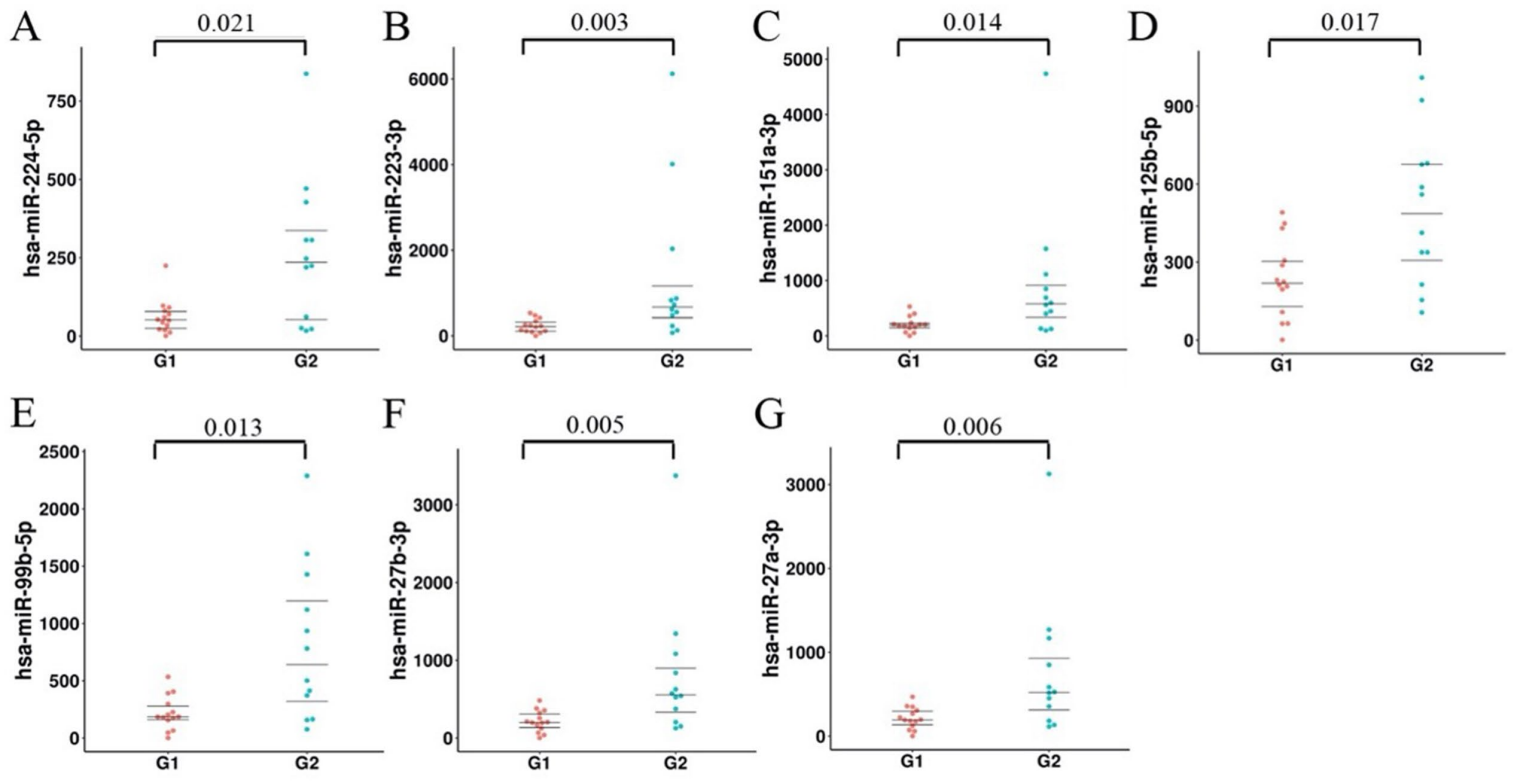
RT-qPCR analysis of differentially expressed miRNAs in plasma exosomes. The values on the vertical axis are all miRNA expression levels (2^−ΔΔCT^). G1 encapsulates the miRNA expression profile in plasma exosomes from patients without brain metastasis, and G2 embodies that from patients with brain metastasis. The expression magnitudes of 7 differentially expressed miRNAs were notably up-regulated in plasma exosomes of lung cancer patients with brain metastases. The orange dots represent the expression level of miRNA in plasma exosomes from patients without brain metastases, while the green dots represent the expression level of miRNA in plasma exosomes from patients with brain metastases.

MiRNA sequencing revealed the top 10 significantly expressed miRNAs in plasma exosomes from lung cancer patients with brain metastases: miR-224-5p, miR-151a-3p, miR-99b-5p, miR27a-3p, miR27b-3p, miR-432-5p, miR-382-5p, miR361-3p, miR-223-3p, miR-125b-5p. RT-qPCR analysis revealed that the expression magnitudes of 7 among the 10 differentially expressed miRNAs were notably up-regulated in plasma exosomes of lung cancer patients with brain metastases, as follows: miR-224-5p, miR-223-3p, miR-151a-3p, miR-125b-5p, miR-99b-5p, miR-27b-3p, miR-27a-3p (Figure 6). The first three exosomal miRNAs with the most significant differences were selected for subsequent diagnostic performance verification, which are miR-223-3p, miR-27a-3p, and miR-27b-3p.

### Metabolomic analysis of short-chain fatty acids in plasma exosomes of advanced lung cancer patients with or without brain metastasis

**Figure 7:**
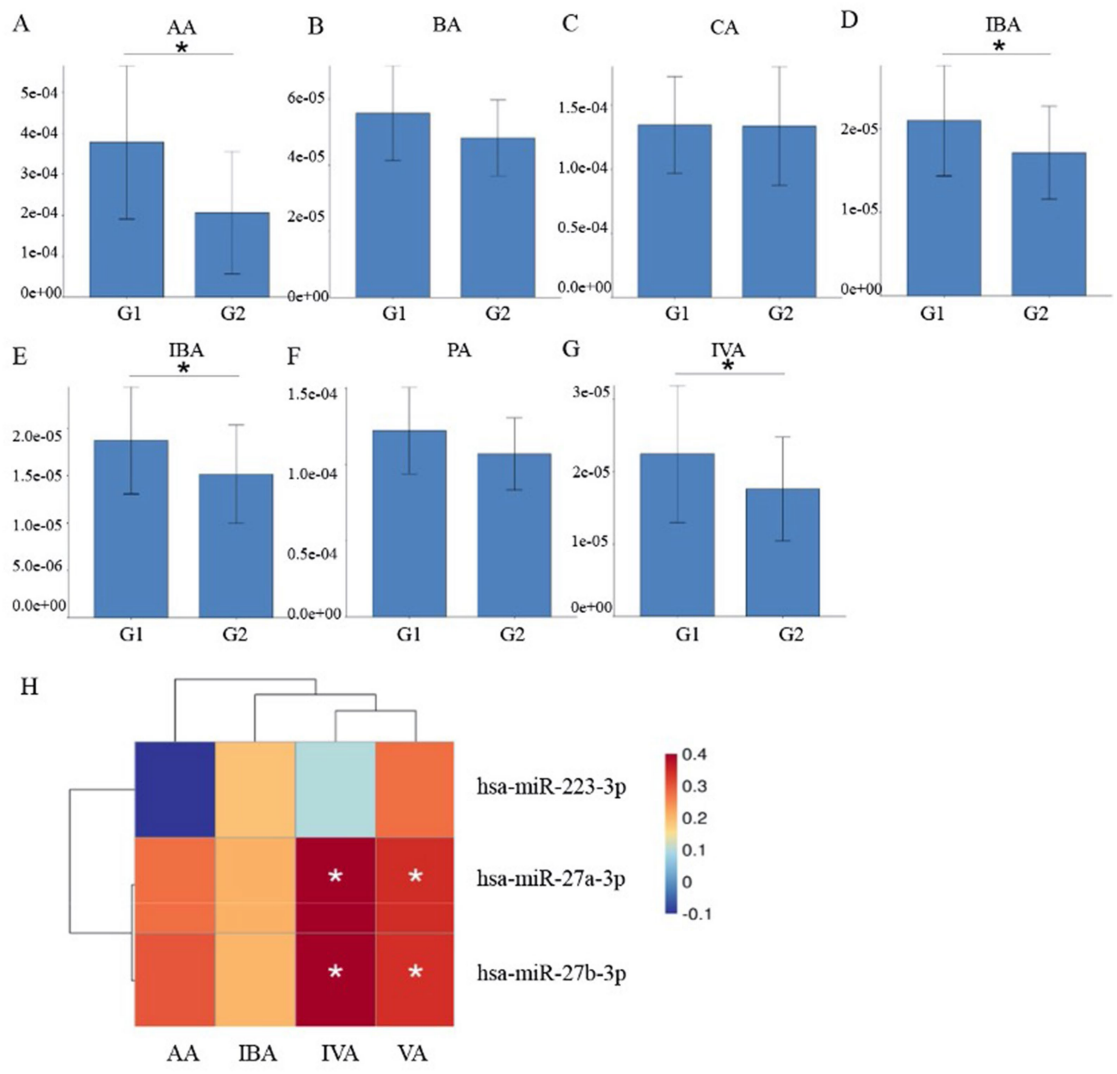
Metabonomic analysis of short-chain fatty acids in plasma exosomes. **(A–G)** Concentrations of various short-chain fatty acids, including acetic acid (AA), butyric acid (BA), caproic acid (CA), isobutyric acid (IBA), isovaleric acid (IVA), propionic acid (PA), and valeric acid (VA). **(H)** Spearman correlation analysis elucidating the relationships between differentially expressed short-chain fatty acids and miRNAs. It revealed that both miR-27a-3p and miR-27b-3p had significant associations with IVA and VA.

As indicated by KEGG pathway enrichment analysis, the butyric acid (BA) metabolism pathway may play a role in regulating lung cancer brain metastasis. BA is a short-chain fatty acid. Consequently, we undertook a metabolomic study of short-chain fatty acids present in plasma exosomes of advanced lung cancer patients, both with and without brain metastasis. The clinical information of the patients was shown in Table 2. In Table 2, “NOS” (not otherwise specified) represents cases where the histological subtype cannot be precisely determined or does not fall into other designated categories. This analysis included acetic acid (AA), propionic acid (PA), isobutyric acid (IBA), BA, isovaleric acid (IVA), valeric acid (VA), and caproic acid (CA). As depicted in Figure 7, the concentrations of IBA, IVA, VA, and AA were markedly elevated in the plasma exosomes of lung cancer patients with brain metastasis. Spearman correlation analysis revealed that both miR-27a-3p and miR-27b-3p had significant associations with IVA and VA.

**Table 2:** Clinical information of patients participating in metabolomic analysis of plasma exosomes.

		Brain metastasis		
Characteristic	Overall	No	Yes	*p*
	50	26	24	
Age [median (IQR)]	65 [60, 69]	65.000 [63, 70]	66 [58, 69]	0.7483
Gender (%)				
Female	14 (28.00)	6 (23.08)	8 (33.33)	0.6229
Male	36 (72.00)	20 (76.92)	16 (66.67)	
Smoke history^a^ (%)				
Mild	7 (14.00)	4 (15.38)	3 (12.50)	0.8393
Moderate	27 (54.00)	13 (50.00)	14 (58.33)	
Severe	16 (32.00)	9 (34.62)	7 (29.17)	
Histological type (%)				
Adenocarcinoma	29 (58.00)	12 (46.15)	17 (70.83)	0.4409
Adenosquamous	1 (2.00)	1 (3.85)	0 (0.00)	
Squamous	6 (12.00)	5 (19.23)	1 (4.17)	
NOS	7 (14.00)	4 (15.38)	3 (12.50)	
Others	7 (14.00)	4 (15.38)	3 (12.50)	
Gene mutation (%)				
EGFR	15 (33.33)	8 (34.78)	7 (31.82)	0.9358
KRAS	4 (8.89)	2 (8.70)	2 (9.09)	
Others	3 (6.67)	1 (4.35)	2 (9.09)	
WT	23 (51.11)	12 (52.17)	11 (50.00)	
Other metastasis (%)				
Bone metastasis	15 (30.00)	6 (23.08)	9 (37.50)	0.369
Liver metastasis	1 (2.00)	1 (3.85)	0 (0.00)	
None	34 (68.00)	19 (73.08)	15 (62.50)	

a Smoking index (SI) = daily smoking quantity × years of smoking. SI ≤ 200: Mild smoking; 200–400: Moderate smoking; ≥ 400: Severe smoking.

### Analysis of the diagnostic performance of plasma exosomal miRNAs and metabolomics for lung cancer brain metastasis

**Figure 8:**
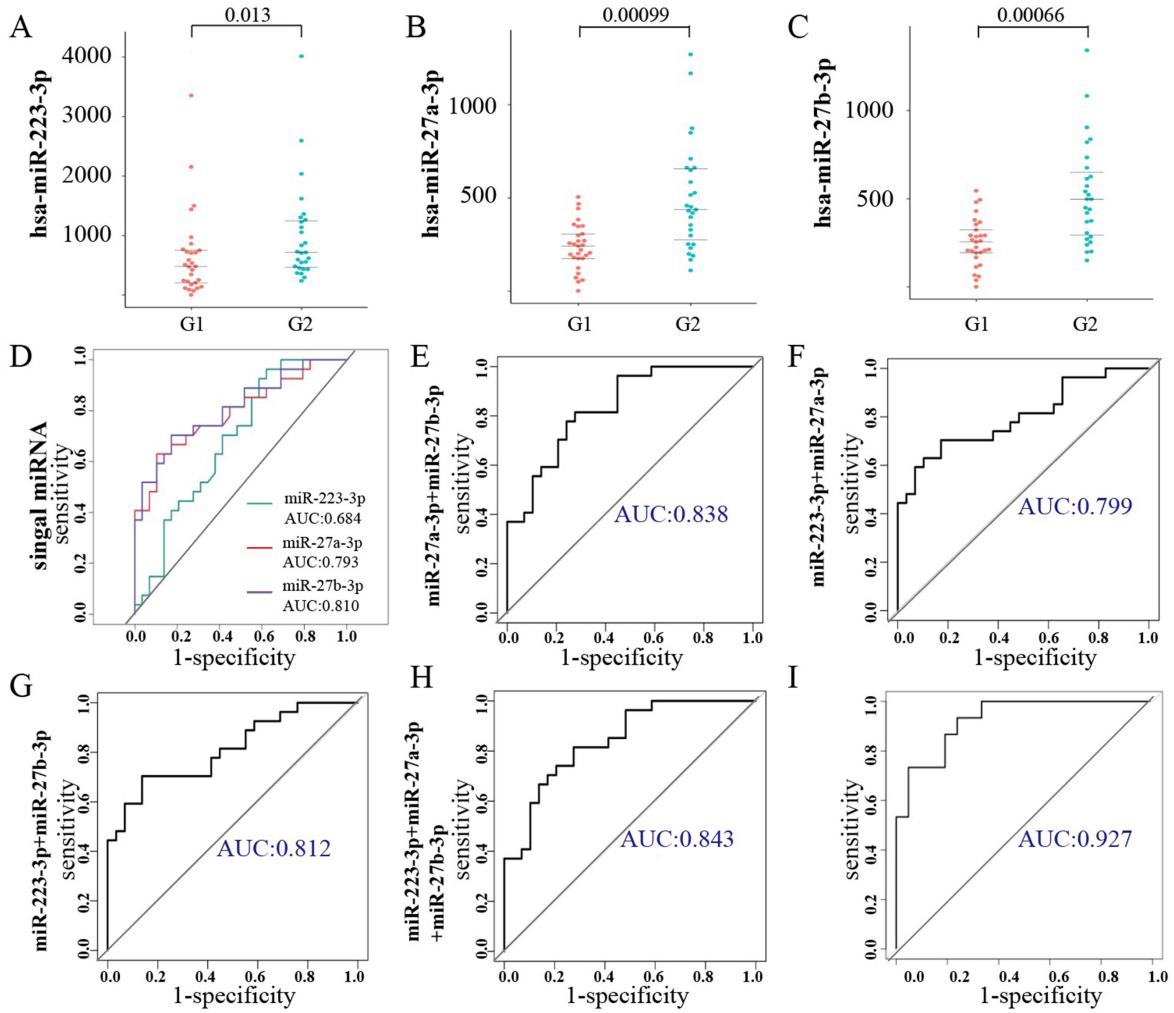
Verification of the diagnostic performance of exosomal miRNAs as biomarkers for lung cancer brain metastasis. **(A–C)** RT-qPCR analysis of miR-223-3p, miR-27a-3p, and miR-27b-3p in patients’ plasma exosomes. **(D–H)** Area under the curve (AUC) values illustrating the diagnostic performance of individual and combined miRNAs as potential biomarkers. **(D)** Calculate the AUC values for miR-223-3p, miR-27a-3p, and miR-27b-3p, respectively. **(E–G)** The pairwise combinatorial analysis was performed on these three miRNAs. **(H)** When all three miRNAs were analyzed together, the AUC reached its peak value. **(I)** Area under the curve (AUC) values of the multi-biomarker model by combining the exosomal miRNAs with metabolic molecules.

In this study, 56 patients with advanced lung cancer were enrolled to collect plasma samples for further validation; the clinical information of patients is detailed in Table 3. RT-qPCR was performed to verify the expression of miR-223-3p, miR-27a-3p, and miR-27b-3p in plasma exosomes, and the results showed that exosomal miR-223-3p, miR-27a-3p, and miR-27b-3p were significantly overexpressed in the plasma of lung cancer patients with brain metastasis (Figure 8). However, they showed no significant association with patient gender, age, or smoking history.

**Table 3:** Clinical information of patients enrolled in verification of the diagnostic performance of exosomal miRNAs.

		Brain metastasis		
Characteristic	Overall	No	Yes	*p*
	56	30	26	
Age [median (IQR)]	65 [56, 68]	65 [56, 69]	66 [57, 68]	0.8179
Gender (%)				
Female	12 (21.43)	5 (16.67)	7 (26.92)	0.5443
Male	44 (78.57)	25 (83.33)	19 (73.08)	
Smoke history^a^ (%)				
Mild	8 (14.29)	5 (16.67)	3 (11.54)	0.8464
Moderate	34 (60.71)	18 (60.00)	16 (61.54)	
Severe	14 (25.00)	7 (23.33)	7 (26.92)	
Histological type (%)				
Adenocarcinoma	39 (69.64)	20 (66.67)	19 (73.08)	0.3293
Adenosquamous	1 (1.79)	1 (3.33)	0 (0.00)	
Squamous	4 (7.14)	4 (13.33)	0 (0.00)	
NOS	4 (7.14)	1 (3.33)	3 (11.54)	
Others	8 (14.29)	4 (13.33)	4 (15.38)	
Gene mutation (%)				
EGFR	13 (25.49)	8 (29.63)	5 (20.83)	0.7562
KRAS	5 (9.80)	2 (7.41)	3 (12.50)	
Others	3 (5.88)	1 (3.70)	2 (8.33)	
WT	30 (58.82)	16 (59.26)	14 (58.33)	
Other metastasis (%)				
Abdominal lymph node metastasis	1 (1.79)	1 (3.33)	0 (0.00)	0.2814
Bone metastasis	17 (30.36)	8 (26.67)	9 (34.62)	
Liver metastasis	3 (5.36)	3 (10.00)	0 (0.00)	
None	35 (62.50)	18 (60.00)	17 (65.38)	

a Smoking index (SI) = daily smoking quantity × years of smoking. SI ≤ 200: Mild smoking; 200–400: Moderate smoking; ≥ 400: Severe smoking.

ROC analysis revealed that the AUC values of miR-223-3p, miR-27a-3p, and miR-27b-3p were 0.684, 0.793, and 0.810, respectively. The subsequent combinatorial analysis of these three miRNAs was performed to determine if they could enhance diagnostic accuracy. As illustrated in Figure 8, the AUC for the combination of miR-223-3p and miR-27a-3p was 0.799, that of miR-223-3p and miR-27b-3p was 0.812, and that of miR-27a-3p and miR-27b-3p was 0.838. When all three miRNAs were analyzed together, the AUC reached its peak at 0.843. This suggests that the collective analysis of these miRNAs can elevate diagnostic precision, with the trio yielding the optimal diagnostic efficacy. Meanwhile, the AUC values of IBA, IA, VA, AA, and their combination analysis were all less than 0.85; we then combined the screened miRNAs with metabolic molecules for diagnostic performance analysis. We found that the AUC value of miR.223.3p + miR.27a.3p + miR.27b.3p + IBA + IVA + VA + AA was the highest at 0.927.

## Discussion

The incidence of brain metastases originating from lung cancer is substantial, and the therapeutic outcomes are often unsatisfactory, leading to significant clinical challenges. At present, exclusively clinical and pathological factors serve to forecast the likelihood of brain metastasis in lung cancer patients. Yet, the existing data on these predictive parameters are inconsistent and lack clinical applicability. Solely relying on conventional clinical and pathological indicators, such as primary tumor status, tumor histology, lymph node status, and patient age, as their risk ratio is small and prognostic factors are unknown (8). There is an urgent need for a more reliable approach to ascertain which patients face the risk of brain metastasis development.

Recently, molecular investigations have underscored the significance of miRNAs, elucidating their role in tumor genesis and progression. Numerous studies have discerned that a myriad of miRNAs play roles in modulating brain metastasis in lung cancer. Elevated expression of miRNA-378 has been detected in NSCLC primary tumor specimens and their corresponding brain metastasis samples. This overexpression has been associated with enhanced cell migration, infiltrative capacity, tumor proliferation, and angiogenesis both *in vitro* and *in vivo*. Consequently, miRNA-378 might aid clinicians in risk stratification for brain metastasis in NSCLC patients (13). Subramani et al. (14) demonstrated that miRNA-768-3p expression decreased in diverse brain metastases relative to their primary tumor counterparts. Zhao et al. (15) reported that compared with 40 primary lung adenocarcinoma cases, miRNA-1471 and miRNA-9 were significantly upregulated in 11 brain metastatic lung cancer specimens, while miRNA-214 and miRNA-145 were less expressed. Augmented levels of miRNA-145 in primary lung adenocarcinoma appeared to deter tumor cell proliferation. miRNAs have been found to influence lung cancer metastasis through multiple molecular signaling pathways. After co-culturing lung cancer cells with astrocytes in vitro, the expression of miRNA-768-3p decreased. This reduction led to increased expression of KRAS and downstream effectors ERK1/2 and BRAF, enhancing the viability of tumor cells and promoting metastasis (14). Chiu et al. (16) reported that ADAM9 could downregulate the expression of miRNA-1 through the activation of the EGFR signaling pathway, boosting the expression of CDCP1 and thereby promoting lung cancer progression. The expression level of miRNA-1 was reduced in primary lung cancer cells but upregulated in lung cancer cells with ADAM9 gene knockout. Furthermore, miRNA-1 demonstrated a negative association with CDCP1 expression and the migratory capacity of lung cancer cells (16). Another study confirmed that miRNA-21 was the target of the signal transducer and activator of transcription 3 (STAT3) in brain metastasis lung cancer cells (17). It was demonstrated that the knockout of the STAT3 gene led to a decrease in the expression levels of the downstream targets of miRNA-21. Both STAT3 and miRNA-21 play roles in regulating tumor cell migration in lung cancer brain metastasis (17, 18).

miRNAs have made significant progress in preclinical and translational studies. However, obtaining lung cancer tissues or brain metastases can be challenging in clinical practice. Over the past few years, the liquid biopsy approach has surfaced as a noninvasive diagnostic modality and has gained prominence in the clinical diagnosis of cancer. Increasingly, studies are discovering that plasma exosomes can serve as novel circulating biomarkers, offering numerous benefits for early diagnosis. Exosomes can shield miRNAs from rapid degradation, and the detection of exosomal contents is more precise. Furthermore, collecting plasma exosomes is straightforward, allowing for ongoing monitoring of molecular markers (19, 20). Currently, no candidate biomarkers in plasma exosome miRNA related to lung cancer brain metastases have been identified.

We performed miRNA sequencing analysis of plasma exosomes from lung cancer patients and identified significantly overexpressed miRNAs in the selected plasma exosomes using RT-qPCR. Our research demonstrated that the expression levels of three miRNAs were markedly upregulated in the plasma exosomes of lung cancer patients with brain metastases: hsa-miR-223-3p, hsa-miR-27a-3p, and hsa-miR-27b-3p. Studies have shown that miR-223-3p is involved in regulating the growth and apoptosis of tumor cells (21, 22), and changes in its expression pattern have been observed in several tumors (23–25). Wang et al. (26) found that exosomal miR-223-3p could promote pulmonary metastasis of breast cancers by targeting Cbx5. Lawson et al. (27) found that miR-223-3p was enriched in lung adenocarcinoma extracellular vesicles, and multiple studies have reported that miR-223-3p can regulate the migration and invasion of lung cancer cells (28, 29). Furthermore, some studies indicated that plasma miR-223-3p could serve as a non-invasive marker for the early diagnosis of lung cancer and is associated with postoperative recurrence in resectable NSCLC (30, 31). MiR-27a-3p has been shown to promote NSCLC formation by regulating ferroptosis (32). Exosomal miR-27a-3p plays a pivotal part in regulating the immune microenvironment of lung adenocarcinoma and correlates with the efficacy of immunotherapy for lung cancer (33). O’Farrell et al. (34) explored a simple blood biomarker (extracellular vesicle miRNAs) to distinguish lung cancer patients and found that plasma exosomal miR-27a-3p was significantly dysregulated in these patients. Research has also shown that miR-27b-3p is involved in regulating the infiltrative capacity, tumor proliferation, and migration of NSCLC cells (35). The expression of miR-27b-3p in exosomes of A549 and A549/DDP cells differs significantly, suggesting that miR-27b-3p may be associated with the resistance of lung cancer cells to cisplatin (36). MiRNAs play a crucial role in cellular signaling pathways, facilitating the expansion of the pre-metastatic niche. Exosomes and exosomal miRNAs regulate the host’s innate and adaptive immune responses by acting on various cells within the immune system (37).

Metabolic disorder is a sign of tumor formation. It is widely acknowledged that metastatic tumor cells require distinct energy, nutrient, and oxygen profiles to outcompete *in situ* cells at the metastatic site and adapt to the microenvironment of local tissues, establishing metastatic colonies. In our study, enrichment analysis of the KEGG pathway for miRNA target genes, differentially expressed in plasma exosomes from patients with or without brain metastasis, showed significant differences in the butyric acid metabolism pathway between the two patient groups. Butyric acid is a short-chain fatty acid and the primary product of intestinal microbial fermentation. It serves as a crucial medium through which the intestinal microbiota regulates systemic energy balance. Its metabolic regulation function includes lipid and glucose metabolism, among other processes (38). The biosynthesis of fatty acids is often elevated in cancer cells to meet the demand for lipids in synthesizing membranes and signaling molecules. Cancer cells often accumulate lipids at higher levels than those in normal cells, typically in the form of lipid droplets (39). We further identified differences in short-chain fatty acid content through metabonomic analysis of plasma exosomes from lung cancer patients, both with and without brain metastasis. The results revealed significant increases in the plasma exosome contents of IBA, IVA, VA, and AA in lung cancer patients with brain metastases. Correlation analysis between the metabolome and miRNAs indicated significant associations of miR-27a-3p and miR-27b-3p with valeric and isovaleric acids. Short-chain fatty acids have been observed to influence the colonization ability of lung cancer cells. Research indicates that low concentrations of butyrate may promote tumor progression and metastasis by upregulating H19 expression and facilitating M2 macrophage polarization and function (40). Butyric acid most effectively enhanced the colonization capability of lung cancer P-29 cells, while propionic and valeric acids had minor effects, and acetic and caproic acids had none (41). Short-chain fatty acids can cross the blood–brain barrier and appear to play a crucial role in maintaining its integrity, which is closely related to the controlled transport of molecules and nutrients from the circulation to the brain and serves a central function in brain development and the preservation of central nervous system homeostasis (42). Currently, no studies have examined the relationship between short-chain fatty acid metabolism and lung cancer brain metastasis. We identified this correlation for the first time, necessitating further research to elucidate the mechanism of short-chain fatty acid metabolism in the onset and progression of lung cancer’s brain metastasis.

In our study, we constructed a diagnostic model using multifactor logistic regression analysis, and ROC analysis confirmed that the three exosomal miRNAs (miR.223.3p, miR.27a.3p, miR.27b.3p) exhibit strong diagnostic performance. When all three miRNAs were analyzed together, the AUC reached its peak at 0.843. To elevate the predictive potency of lung cancer brain metastasis, we constructed a multi-biomarker model by combining the exosomal miRNAs with metabolic molecules for diagnostic performance analysis, with an AUC value of 0.927, reaching the highest level (miR.223.3p + miR.27a.3p + miR.27b.3p + IBA + IVA + VA + AA). Our biomarkers and their combination may contribute to early detection of lung cancer patients with brain metastases and early intervention, which is beneficial for the comprehensive management of lung cancer.

### Limitations of the study

Owing to limitations in time and resources, the sample size for this study is not sufficiently large, which may lead to potential selection bias.

## Conclusion

The plasma exosomal miR-223-3p, miR-27a-3p, and miR-27b-3p in patients with advanced lung cancer are closely related to brain metastasis and can serve as biomarkers for predicting lung cancer brain metastasis. Differences exist in the plasma exosomal short-chain fatty acid metabolism between patients with and without brain metastasis, and these differences are associated with plasma exosomal miR-27a-3p and miR-27b-3p. The regulatory relationship between them requires further investigation. The combined analysis of the exosomal miRNAs (miR-223-3p, miR-27a-3p, miR-27b-3p) and the metabolic molecules (IBA, IVA, VA, AA) offers improved diagnostic performance. These findings have potential as a valuable reference for the early clinical prediction and management of lung cancer patients with brain metastasis.

## Data Availability

The data reported in this paper have been deposited in the OMIX, China National Center for Bioinformation / Beijing Institute of Genomics, Chinese Academy of Sciences, with the accession number OMIX013392, project PRJCA051244. The URL is https://ngdc.cncb.ac.cn/omix/release/OMIX013392.
